# Staff Perspectives on Daily Board Rounds and Weekly Multidisciplinary Reviews in Psychiatric Inpatient Wards in Sheffield – Phase 1

**DOI:** 10.1192/bjo.2026.11609

**Published:** 2026-06-30

**Authors:** Nabil Haddou, Ann Panjikkaran, Marin Marquez, Nicoletta Lekka

**Affiliations:** Sheffield Health Partnership University NHS Foundation Trust, Sheffield, United Kingdom

## Abstract

**Aims:**

Acute inpatient services are experiencing increasing pressure on bed capacity, alongside a growing need for efficient and sustainable use of resources. As services transition towards the NHS ‘Home First’ approach, there is a need to critically review existing practices to further optimise patient flow and reduce discharge delays.

This service evaluation looked at how three acute general adult psychiatric wards in Sheffield utilised brief daily board meetings referred to as ‘Purposeful Inpatient Admissions’ (PIPA) meetings, and weekly multidisciplinary team (MDT) meetings. Phase 1 of the project aimed to gain an understanding of staff views on the existing daily PIPA/weekly MDT model. These findings will inform the development of the new ‘Daily Planning Meeting’ model and provide a meaningful comparator for phase 2 of the service evaluation following implementation.

**Methods:**

An anonymised baseline survey was distributed via Microsoft Forms to staff across the three wards to capture multidisciplinary perspectives. The 21-question survey included Likert-scale and free-text items exploring views on the current model, including workload, communication, service user experience and suggestions for improvement. It remained open for three weeks between November and December 2025. Data were exported to Excel for quantitative and qualitative analysis.

**Results:**

Forty-six staff members responded, with nurses and medics comprising 63%, reflecting ward workforce composition. 52% of respondents found the current PIPA/MDT model ‘somewhat helpful’. 70% reported that PIPA/MDT ‘sometimes’ identified clear care goals. 57% thought that the current model ‘sometimes’ improved relationships with patients whilst 67% felt that it ‘sometimes’ enabled patient and family involvement. 9% of respondents felt the current model ‘never’ supported enthusiasm or engagement in patient-centred care. Most respondents agreed all professional roles should attend MDTs regularly.

Thematic analysis highlighted perceived benefits of the current model which included structured multidisciplinary collaboration, task prioritisation and shared decision making. However, significant challenges were identified. Both meetings were frequently described as time consuming. Communication gaps within and across teams, limited community team involvement and inconsistent patient and family engagement were recurrent themes. Staff advocated for clearer documentation processes, enhanced patient involvement and streamlined meeting structures as ways to improve the current model.

**Conclusion:**

Phase 1 identified key strengths and limitations of the PIPA/MDT model. Recommendations include defining professional roles and responsibilities, improving task allocation and documentation as well as protecting time for meaningful patient engagement. Early involvement of community teams and encouraging patient and family participation are central to supporting the NHS Home First approach and improving patient flow.

